# The Potential of Peptide-Based Inhibitors in Disrupting Protein–Protein Interactions for Targeted Cancer Therapy

**DOI:** 10.3390/ijms26073117

**Published:** 2025-03-28

**Authors:** Alexandra L. Afonso, Catarina T. Cavaleiro, Miguel A. R. B. Castanho, Vera Neves, Marco Cavaco

**Affiliations:** 1 Gulbenkian Institute for Molecular Medicine, Av. Prof. Egas Moniz, 1649-028 Lisboa, Portugal; alexandra.afonso@gimm.pt (A.L.A.); catarina.cavaleiro@gimm.pt (C.T.C.); or macastanho@medicina.ulisboa.pt (M.A.R.B.C.); 2 Faculdade de Medicina, Universidade de Lisboa, Av. Prof. Egas Moniz, 1649-028 Lisboa, Portugal

**Keywords:** peptides, protein–protein interactions (PPIs), OncoPPIs, targeted cancer therapy, peptide-based inhibitors

## Abstract

Protein–protein interactions (PPIs) form an intricate cellular network known as the interactome, which is essential for various cellular processes, such as gene regulation, signal transduction, and metabolic pathways. The dysregulation of this network has been closely linked to various disease states. In cancer, these aberrant PPIs, termed oncogenic PPIs (OncoPPIs), are involved in tumour formation and proliferation. Therefore, the inhibition of OncoPPIs becomes a strategy for targeted cancer therapy. Small molecule inhibitors have been the dominant strategy for PPI inhibition owing to their small size and ability to cross cell membranes. However, peptide-based inhibitors have emerged as compelling alternatives, offering distinct advantages over small molecule inhibitors. Peptides, with their larger size and flexible backbones, can effectively engage with the broad interfaces of PPIs. Their high specificity, lower toxicity, and ease of modification make them promising candidates for targeted cancer therapy. Over the past decade, significant advancements have been made in developing peptide-based inhibitors. This review discusses the critical aspects of targeting PPIs, emphasizes the significance of OncoPPIs in cancer therapy, and explores the advantages of using peptide-based inhibitors as therapeutic agents. It also highlights recent progress in peptide design aimed at overcoming the limitations of peptide therapeutics, offering a comprehensive overview of the current landscape and potential of peptide-based inhibitors in cancer treatment.

## 1. Introduction

Cancer is considered a significant societal, public health, and economic problem in the 21st century, being the second most common cause of death. Accounting for one-fifth of all recorded deaths worldwide, it has resulted in 9.7 million deaths in 2022 along with the diagnosis of 20 million cases [1]. Currently, lung and breast cancer are the most commonly occurring cancers worldwide, accounting, for 12.4% and 11.6% of total new cases, respectively. This is followed by colorectal (9.6%), prostate (7.3%), and stomach cancer (4.9%) [2].

Treatments such as chemotherapy, surgery, and radiation therapy have long been the cornerstone of cancer management. Despite having demonstrated great efficacy in reducing tumour volume and prolonging survival in some cancer patients, they possess significant side-effects due to their non-specific nature, associated with the inhibition of important metabolic pathways in non-cancer cells. Additionally, they often lose their therapeutic use owing to drug resistance and lack of tumour selectivity [3,4,5].

Targeted cancer therapies have become dominant in novel drug development as they offer a more refined approach to treatment [6]. Unlike the traditional treatments, which can result in widespread damage to both cancerous and healthy cells, targeted therapies are designed to selectively interfere with specific molecular targets that drive tumour growth and/or progression. By honing in on key proteins, receptors, and signalling pathways that are dysregulated in cancer cells, these therapies disrupt the underlying mechanisms driving malignancy while sparing healthy cells and tissues [7]. On that account, targeted therapies offer improved efficacy with fewer side effects, having merit in selectivity, efficacy, and tolerability compared to traditional treatments [8,9,10].

The current predominant forms of targeted therapy are monoclonal antibodies (mAbs) and small-molecule drugs (SMDs) [11,12]. Therapeutic peptides have emerged as promising alternatives to standard approaches due to their unique physicochemical properties. Composed of a series of a well-ordered amino acids, usually with molecular weights (MWs) ranging from 0.5 to 5 kDa, peptides have several important advantages over small molecules (e.g., high specificity and affinity for their targets, minimal drug-drug interaction, and lower toxicity) and antibodies (e.g., lower toxicity and immunogenicity) [13]. Moreover, peptides are characterised by their rapid and cost-effective synthesis, as well as their ease of modification. An additional benefit is that peptides generally do not accumulate in key organs, such as the kidneys or liver, thereby mitigating the potential for adverse effects [14].

Following the approval of the first therapeutic peptide, the insulin hormone in 1923, the development of therapeutic peptides has been progressively expanding [15]. In 2022, they accounted for 5% of the global pharmaceutical market, representing a field of growing interest [16]. Approximately 100 therapeutic peptide drugs have been approved, with more than 200 undergoing clinical development (Table 1).

A promising area of peptide-based therapeutics is the targeting of protein–protein interactions (PPIs). PPIs comprise a vast and complex network, the interactome, orchestrating fundamental cellular processes ranging from gene regulation to signal transduction and metabolic pathways [56,57]. The dysregulation of this interactome has been vastly implicated in the onset and progression of various disease states [58,59,60]. In cancer, these aberrant protein–protein interactions, termed OncoPPIs, are pivotal in driving oncogenesis [61,62]. Thereby, the inhibition of this specific subset of PPIs was established as a promising strategy for targeted cancer therapy [63,64].

Over the past decade, significant strides were witnessed in targeting of challenging PPIs with SMDs. Nonetheless, peptide-based inhibitors can address certain challenges that have remained elusive to their small molecule counterparts, such as improved specificity and enhanced target binding [65]. Indeed, peptide-based inhibitors targeting OncoPPIs have shown quite promising results, highlighting their potential as therapeutic agents, which will be further explored throughout this review.

This review outlines the key characteristics and challenges associated with targeting PPIs, as well as the importance of targeting cancer-specific protein interactions (OncoPPIs) for effective cancer therapy. It also delves into the benefits of opting for peptide-based inhibitors, followed by exploring recent advancements in peptide design to circumvent the inherent limitations associated with peptide-based therapeutics. Finally, the review offers insights into the current landscape of peptide-based inhibitors as viable options for cancer therapy.

## 2. Protein–Protein Interactions: Specific Targeting and Challenges

Proteins are one of the building blocks of the cell and make up nearly half of its dry mass. They rarely act individually, instead, their role arises from the interaction among multiple proteins and other biomolecules [66,67]. PPIs are fundamental processes governing all cellular activities, including signal transduction, gene regulation, and metabolic pathways [56]. They involve the binding of two or more protein molecules through specific regions and display a range of heterogeneities in macromolecular structures, forming protein dimers, multicomponent complexes, or long chains. These interactions are dynamic and occur with different affinities and durations, allowing proteins to interact transiently or persistently depending on the cellular context [57,68,69]. PPIs make up a large-scale and complex network termed the interactome [68,70,71]. Several computational and experimental approaches have been developed to determine and understand PPI networks in cells, with hundreds of thousands of human protein interactions being characterised to date, which represents a significant repertoire of targets for therapeutic discovery [72]. Even so, these only consist of fewer than 5% of the total existent protein interactions [73]. Hence, PPIs hold vast untapped potential for developing new therapeutic targets given their pivotal role in numerous physiological processes.

Over the past few years, PPIs have undergone a significant transformation in our understanding [74,75]. Once deemed “undruggable”, PPIs have now transitioned into a “yet to drug” category, opening up new avenues for research. This shift in perception has been fuelled by the emergence of high-quality PPI system crystal structures, which have provided valuable insights in the field of PPI inhibitor drug development. However, despite these advancements, the biophysical and biochemical limitations of PPIs continue to pose challenges in drug discovery [76,77].

One of the major difficulties for PPI targeting is their large, shallow, and featureless interfaces [78,79]. Not only do they tend to be flat, containing few grooves or pockets for proper binding, they are also highly hydrophobic [79,80]. Indeed, retrospective studies demonstrated that the PPI interface is mainly occupied by 56% non-polar groups, 29% by polar groups, and 15% by charged groups [81]. This unfavourable architecture makes it difficult for effective ligand binding and challenges the design and optimisation of drug molecules [82]. Previous crystallography and modelling studies have shown that the protein binding interface is generally wide and smooth, and its surface area ranges between 1500 and 3000 Å^2^ [83,84]. Furthermore, the amino acid residues involved in PPIs are either continuous or discontinuous in their respective protein structures, leading to high affinity and tight interactions between PPI partners [85]. This also poses a great challenge for ligands to compete for binding orthosterically, which refers to binding at the active site whilst competing with the natural substrate, thus hampering PPI inhibitor design [65].

A critical advancement in understanding PPIs was the understanding that the interactions driving the affinity of a pair of proteins are not evenly distributed across their surfaces. Rather, specific residues or regions, called hot spots, are largely responsible for driving the binding [86,87]. The introduction of the concept of “hot spots” has challenged the perception of PPIs as undruggable targets [65,72].

Hot spots can be explored by a process called alanine scanning or hot-spot analysis, which checks the effect of sequentially mutating amino acid residues to alanine (Ala) (or mutating Ala residues to glycine (Gly)) on the affinity of a pair of proteins. So, within the PPI interface, when a residue mutated to Ala causes a PPI binding energy drop of more than 2 kcal/mol, it is defined as a hot spot [86]. It has been established that the area of hotspots is about 600 Å^2^ and that they are mainly located at or in the proximity of the PPI interface [83,88]. Furthermore, analysis of alanine scanning data indicates that tryptophan (Trp), tyrosine (Tyr), and arginine (Arg) are more likely to appear as hot-spot residues, and, to a lesser extent, the polar residues aspartic acid (Asp) and histidine (His) are also enriched [87,89]. Hot-spot residues have since provided crucial insights into the development and design of PPI inhibitors. Considering the relatively large size of the PPI interfaces, the molecule of interest must act on these hot-spot regions to disrupt whole PPIs. Hence, hot spots offer more precise and well-defined drug targets than the broad and extensive protein interaction surfaces. By targeting these residues within a localized region, inhibitors can effectively avoid competition with high-affinity protein binding effectors, while disrupting overall PPI complexes and exerting therapeutic effects.

## 3. Targeting PPIs as an Anticancer Strategy

Given the importance and intricacy of PPIs, it is unsurprising that these networks can be altered in disease states. Herein, the well organised and precisely regulated PPI network undergoes disruption and becomes hijacked or reprogrammed. This dysregulation has been well documented in the onset and progression of various conditions [58,59,60].

Cancer cells have significantly altered PPIs compared to healthy cells [61,85]. These interactions, known as OncoPPIs, primarily consist of the interaction network of oncoproteins [62]. OncoPPIs, a subset within the broader classification of PPIs, are not just an anomaly but a central player in all stages of oncogenesis, influencing processes such as cell proliferation, survival, inflammation, invasion, and metastasis, thereby constituting a hallmark of cancer [61,85,90].

OncoPPIs exhibit abnormal expression patterns, which can include overexpression, under-expression, or mutations compared to normal cells [63]. Importantly, research on oncogenic protein–protein interfaces has revealed that cancer-associated proteins have smaller, more planar, more charged, and less hydrophobic binding sites compared to non-cancer proteins. This conformation indicates that OncoPPIs display high specificity for other cancer-related PPIs, preferentially interacting with cancer-associated proteins, thus making them challenging targets [64]. Nonetheless, it is recognised that strategies aimed at disrupting this subset of PPIs hold significant promise for effective targeted cancer therapeutic development, inspiring a new era in cancer research [61].

The targeting of OncoPPIs starts with the deciphering of cancer-specific interactomes. Advances in technology and proteomics have facilitated large-scale, high-throughput screening studies of PPIs. A fast-growing number of PPIs detected through diverse methodologies are now catalogued in large PPI databases, offering a comprehensive view of the human interactome [91,92,93]. Notably, publicly available databases such as Biogrid, Bioplex, IntAct, MINT, and HIPPIES update and curate human protein interactions, enhancing the understanding of cellular signalling networks [94,95,96,97,98,99].

However, despite the auspicious role of OncoPPIs as druggable cancer targets, their large-scale experimental identification remains challenging. The inherent complexities in identifying and characterising OncoPPIs have greatly hindered mapping efforts [94]. Thus, currently available resources to explore oncogenic PPI networks are still limited and require improvement. Databases like PINA merge cancer transcriptomics and proteomics information with PPI datasets, yet they do not differentiate between normal protein interactions and OncoPPIs [100]. A newer platform, the OncoPPI Portal, has been introduced based on the characterisation of around 3500 PPIs tested for a set of lung-cancer related proteins, resulting in a network of high-confidence direct PPIs [62,91]. Still, studies to build reference OncoPPI maps for various cancer types have not been undertaken [94]. These databases would undoubtedly have significant potential in the development and discovery of novel targets for cancer therapy. The deciphering of cancer-specific interactomes would undoubtedly aid in uncovering new mechanisms of oncogenic signalling for therapeutic interrogation.

Hence, similarly to PPIs, targeting OncoPPIs has emerged as a promising strategy for cancer therapeutic intervention. Understanding the mechanisms underlying PPIs and devising strategies to efficiently modulate these interactions becomes critical for optimising and proposing new effective cancer therapies.

## 4. Current Approaches to PPI Inhibition

Presently, PPI inhibition involves the use of small molecule inhibitors, peptide-based inhibitors, and small biopharmaceutical proteins (e.g., nanobodies) [101]. Small molecule inhibitors have long been the dominant strategy for PPI inhibition, with remarkable advances in handling challenging PPI targets being witnessed in the last decade [102]. Particularly, venetoclax was the first and currently the only approved small-molecule B-cell lymphoma-2 (Bcl-2) inhibitor effective in treating chronic lymphocytic leukaemia (CLL) [103]. Notably, afatinib was the first Epidermal Growth Factor Receptor (EGFR) inhibitor approved by the Food and Drug Administration (FDA) for lung cancer [104]. Sotorasib, for non-small cell lung carcinoma (NSCLC) patients, was also the first targeted drug for treating Kristen Rat Sarcoma Viral oncogene homolog (KRAS) gene mutation [105].

Offering a diverse array of chemical structures and pharmacological properties, SMDs are characterised by their small size compared to other therapeutics (<500 Da), great oral bioavailability, and ability to cross cell membranes [78,106]. The convenience of oral administration, which can enhance patient compliance, coupled with their small molecular size, which enables facile cell membrane penetration to target intracellular molecules, has made small molecule inhibitors the traditional focus for PPI targeting [107,108]. However, despite their numerous advantages, small molecule inhibitors face several challenges in effectively modulating PPIs. Primarily, their small size greatly restricts their interaction with the extensive interfaces characteristic of PPIs. Typically occupying only 300–1000 Å^2^ of the contact area, small molecule inhibitors cover only a fraction of the contact area required for effective inhibition [79]. Additionally, SMDs have an inherent lack of specificity. This generally leads to off-target interactions and adverse effects, thus restricting their clinical use [109].

While small molecules have historically dominated the field of PPI inhibition, peptide-based inhibitors later emerged as compelling alternatives, currently comprising approximately 40% of all PPI inhibitor cases [84].

Peptide-based inhibitors offer distinct advantages over their small molecule counterparts. Namely, peptides are rationally designed based on the sequences that mediate PPIs, resulting in peptides that resemble endogenous ligands and that are able to target critical binding surfaces. Unlike small molecules, peptides also possess a larger size and more flexible backbones. Being classified as medium-sized inhibitors with an MW of 0.5 to 5 kDa, peptides can effectively engage with the expansive interfaces characteristic of PPIs, hence being more effective than small molecule inhibitors. Furthermore, peptides exhibit a more favourable selectivity for their target, affordable synthesis availability, and lower toxicity profiles, positioning them as promising candidates for targeting PPIs [13,110,111,112]. Interestingly, it has also been estimated that 15–40% of all PPIs are mediated by short linear peptides, emphasizing the significance of peptides in modulating these interactions [113].

Despite their advantages, peptide-based inhibitors face challenges that can hinder their therapeutic use. In general, peptides usually lack well-defined secondary or tertiary structures, making them susceptible to proteolytic degradation [114]. The absence of structural stability also translates into a short half-life and fast elimination, which poses challenges in maintaining therapeutic concentrations of peptide drugs, often requiring frequent dosing to sustain their effectiveness [13,115,116]. Additionally, peptides have large flexibility in the absence of binding, and thus can promote a significant entropy-related penalty upon binding [117].

Overcoming the inherent limitations of peptides has been a longstanding challenge in the design and application of peptide-based inhibitors. Historically, peptides were often disregarded as lead therapeutics due to their disadvantages. In response, a plethora of solutions has been proposed to address these drawbacks, aiming to improve the properties of peptide-based compounds and expand their applications in therapeutic fields. These advances have propelled peptide-based therapeutics to become increasingly viable for pharmaceutical applications, driving their growth in industrial pipelines [116].

## 5. Chemical Strategies to Overcome Peptide-Based Inhibitor Limitations

**Figure 1:**
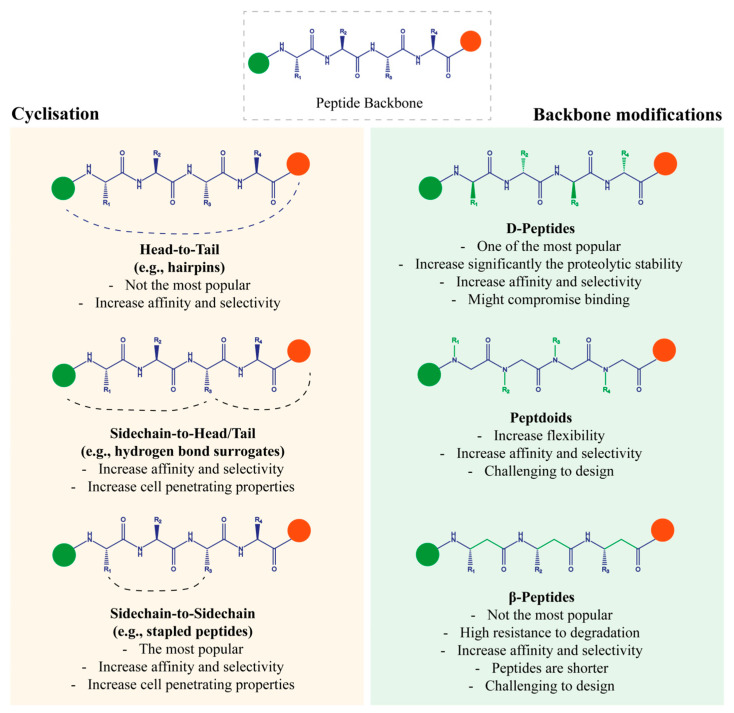
Main strategies applied to peptide-based inhibitors for protein–protein interactions (PPIs) in cancer therapy.

Enhancing peptide stability and mitigating their sensitivity to proteolysis are the main challenges for peptide-based inhibitor design and development. Chemical modifications allow the tailoring of peptides to successfully enhance their role. They aim at constraining the peptide’s secondary structure into a stable and specific conformation to improve its biophysical properties. Noteworthy strategies include cyclisation and backbone alteration, which have emerged as pivotal strategies to circumvent these drawbacks (Figure 1). These advancements have paved the way for developing peptide-based inhibitors with improved inhibitory activity and pharmacokinetic (PK) properties compared with non-modified peptides. The implementation of these techniques, having also earned the title of peptidomimetics, has already obtained considerable success in PPI inhibition, particularly in the cancer field.

### 5.1. Cyclisation

Cyclisation of peptides has gathered extensive attention due to their acquired properties. Cyclic peptides have been shown to enhance bioactivity and lower toxicity, with improved affinity for their targets [118,119,120,121,122,123]. Cyclising a peptide aims to rigidify its structure in the active conformation. Numerous strategies, such as hairpins, stapling, and hydrogen bond surrogates, were developed to stabilise turns, helices, and extended conformations within peptide scaffolds [65,124]. The overall strategy is elucidated according to the conformation of the native ligand, often being based on the stabilisation of the peptide secondary structure [124,125].

#### 5.1.1. Hydrogen Bond Surrogates (Sidechain-to-Head/Tail)

Hydrogen bond surrogates (HBSs) consist of replacing with a covalent bond the interaction between the N-terminal i to i + 4 amino acid residues, thus generating an a-helix conformation [126,127,128]. The conformation of the HBS helix greatly improves the overall structural stability and rigidity of the peptides, further augmenting their resistance to proteolysis [118]. Moreover, these compounds have increased cell penetrating properties compared to their linear counterparts [127,129]. Markedly, an HBS can mimic the backbone hydrogen bond without manipulating the molecular recognition surface of the helices. It retains the sidechain functionalities of the amino acids, similar to native sequences, thus making the remaining sidechains available for target recognition [118,130]. Various covalent linkers have been incorporated as HBSs, such as hydrazone, thioether, olefin, disulfide, and alkyl (ethylene and propyl) [128,131,132,133,134].

- Bcl-xL

**Figure 2:**
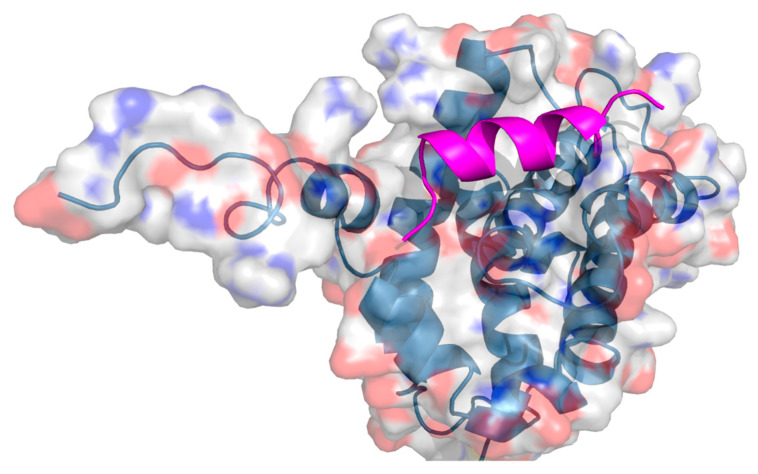
Crystal structure of a bcl-xl/bak and hydrogen-bond surrogate (HBS) peptide inhibitor [135]. The receptor is displayed with a transparent surface coloured element. The HBS peptide is presented in the graphic representation in magenta. PDB: 1BXL.

The first biologically relevant HBS-constrained peptide was reported by Wang et al. B-cell lymphoma-extra-large (Bcl-xL) is an antiapoptotic protein that regulates cell death by binding the α-helical BH3 domain of a family of proapoptotic proteins (including Bak, Bad, Bid, and Bax), with this PPI being severely impaired in a multitude of cancers. Two olefin HBS-constrained peptides were generated based on the conserved α-helical Bcl-xL interacting domain of Bak BH3. They were found to be extremely stable towards proteolytic cleavage and exhibited around a 60-fold increase in stability compared to the original peptide [135] (Figure 2).

- p53/MDM2

The p53/Mouse Double Minute 2 (p53/MDM2) PPI has long been as an attractive target for cancer therapy owing to its major role in cancer progression. Disrupting the MDM2–p53 interaction is a therapeutic strategy that aims to prevent degradation of the p53 tumour suppressor in cancer cells [136]. An olefin HBS-stabilised α-helical peptide inhibitor was developed with a two-fold higher affinity, effectively disrupting the PPI [137]. A thioether-linked HBS-peptide was also developed against this PPI, having a similar affinity value [134].

- Ras/sos

The interaction between Rat sarcoma virus (Ras) and Son of sevenless (Sos), which is involved in the receptor tyrosine kinase (RTK) signalling pathway, also poses a great therapeutic target [138]. This oncogenic PPI was successfully inhibited by an HBS-constrained peptide displaying an affinity 10 times greater than the control peptide [139].

#### 5.1.2. β-Hairpin (Head-to-Tail)

The designing of hairpin peptides requires the identification of the epitopes that are involved in PPIs. These are then transferred on semi-rigid macrocyclic templates of hairpin loop sequences [130,140]. β-hairpins require a turn-inducing unit, with d-Pro-l-Pro being the most commonly used fragment for this purpose [141,142]. This cyclisation is performed through the coupling of N- and C- termini, forming a closed loop that connects both termini of the peptide, hence the head-to-tail denomination [143]. However, some other methods have been reported, such as the reactivity of sidechains, including disulfide formation, azide–alkyne cyclo-addition, and Trp–Trp crosslinking [130,144,145]. Despite not being one of the most popular modifications in peptide-based inhibitors, the hairpin conformation also seems to exhibit high affinity and selectivity for the selected targets [142,146,147].

- p53/HDM2

Fasan et al. designed a β-hairpin that mimics the α-helix of p53, inhibiting the p53– human MDM2 (HDM2) interaction. The cyclic antiparallel peptide uses a dimer of d-Pro-l-Pro as a turn inducer [142]. Still, in p53 targeting, a disulfide-rich backbone-cyclised polypeptide showed efficient inhibition of interactions between p53 and its inhibitors MDM2 and MDMX, reducing cancer cell proliferation in vitro and reducing the tumour growth rate in mice [148]. In a recent development, β-hairpins designed by Nadal-Bufi et al. have successfully inhibited LDH5 activity in vitro with a low micromolar range and more efficiently than the already existing small molecule inhibitor.

- p53/MDM2

Voelz and co-workers have recently performed molecular dynamics simulations to examine the role of preorganisation of a cyclic β-hairpin in the binding mechanism and affinities for MDM2, highlighting the importance of molecular simulation work in the discovery of peptides with enhanced binding affinities [149].

- PD-1/PD-L1

**Figure 3:**
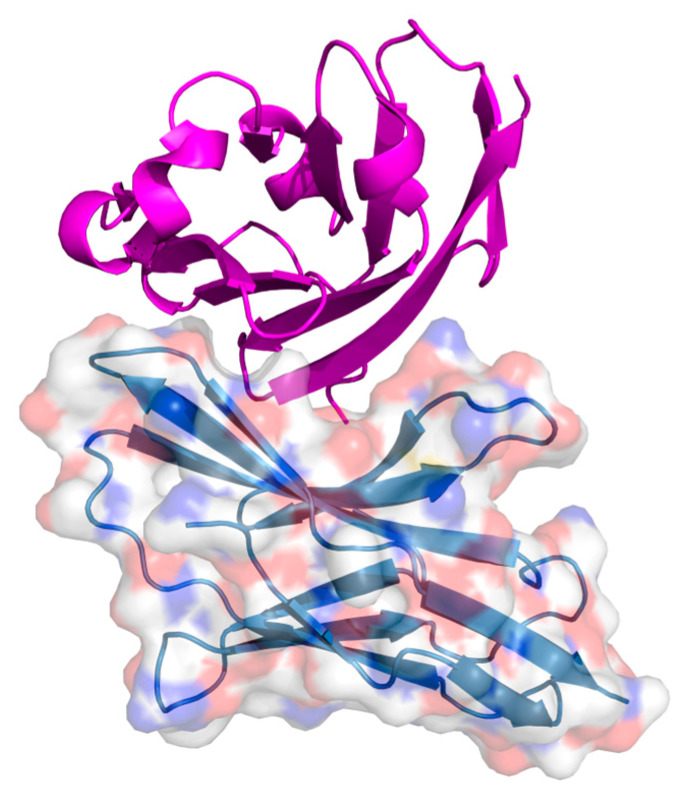
Crystal structure of the hairpin peptide (magenta) interacting with PD-1 [147]. The PD-1 receptor is depicted with a transparent surface coloured element. The peptide is magenta-coloured in the graphic representation. PDB: 4ZQK.

Wang et al. also synthesised a series of peptide-based inhibitors based on a continuous sequence of 14 amino acids from PD-L1 and performed modifications to form a hairpin structure. The hairpin structure with increased stability also improved the affinity of inhibitors to PD-1 and increased IL-2 secretion, showing that peptide inhibitors may be a useful approach to block the interaction between PD-1 and PD-L1 [147] (Figure 3).

- BRCA1-BRCT

Combining in silico design and virtual screening tools, two cyclic β-hairpin peptides were designed as mimetics of the interaction between the Breast Cancer Type 1 and BRCA1 carboxyl-terminals (BRCA1-BRCT domain). These will proceed with experimental validation due to their affinity and selectivity towards the target, once more emphasizing the role of computational tools in the discovery and optimisation of peptide-based inhibitors [150].

#### 5.1.3. Stapled Peptides (Sidechain-to-Sidechain)

Intramolecular sidechain-to-sidechain crosslinking or stapling is considered a powerful tool for perturbing PPIs and is perhaps the most popular [130]. The design is based on the tethering of sidechains at i and i + 3, i and i + 4, or i and i + 7 residues via a chemical linker to restrict the flexibility and maintain the α-helix structures of the peptides. In most cases, i, i + 4 or i, i + 7 hydrocarbon staples that contain 8 or 11 carbon atoms, respectively, are formed [151,152]. Importantly, stapled peptides are the most common peptide inhibitors of PPIs, as they restore and enhance peptides’ natural α-helical structures [151,153,154]. This allows them to bind large and flat surfaces efficiently and specifically, which is required in most PPIs. However, because they often adopt a much wider range of 3D structures than α-helices, there is no optimal staple for cyclic peptides in general. To apply this strategy, a systematic exploration of crosslink positions and geometries is required to achieve peptides with enhanced functional properties [155,156]. Most common linkages that are incorporated into the sidechains include disulfide and lactam bridges, ring-closing metathesis, cysteine crosslinking, C-H activation, and thioether linkages, although others have been reported. Recently, the adding of α-Amino Acid-derived peptidyl staples has also shown improved enzymatic stability and binding affinity [157,158,159,160,161]. Overall, stapling generally enhances binding through hydrophobic interactions with the target molecule, with properly designed stapled peptides exhibiting benefits such as enhanced proteolytic stability and binding, stabilised structure, and, in some cases, cell permeability [154,162].

- p53/MDM2

Numerous studies highlight the use of stapled peptides in cancer treatment, with p53 and MDM2 being a particularly common target [163]. For this inhibition, an all-hydrocarbon strategy has been greatly reported [121,164,165,166]. Bernal et al. designed 10 stapled peptides using an all-hydrocarbon stapling strategy. The preferred peptide, SAH-p53–8, can transmigrate through cell membranes to induce apoptosis in tumour cells [162]. Wang et al. went on to further mutate this peptide by using a thiolene staple. Compared to unstapled peptides, these demonstrated a higher α-helicity degree [167]. More recently, peptides targeting this PPI with Diels–Alder cyclisation and Ugi-MCR have also been reported [168,169].

- MDM2/MDM4

**Figure 4:**
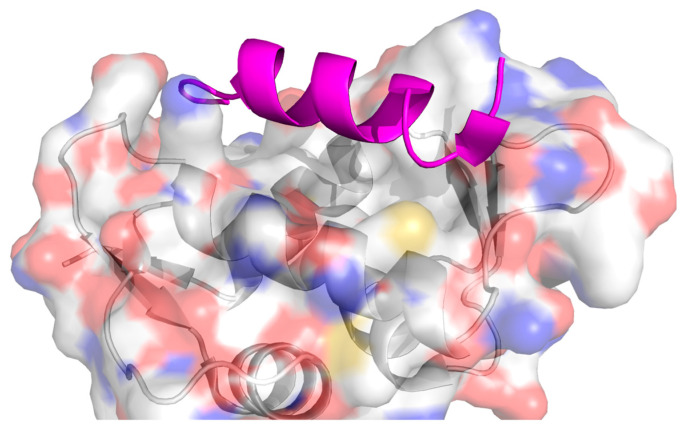
Crystal structure of the stapled peptide ALRN-6924 interacting with MDMX [170]. The MDMX receptor is displayed with a transparent surface coloured element. The stapled peptide is presented in magenta as a graphic representation. PDB: 8GJS.

Sulanemadlin (ALRN-6924) is the first cell-permeating α-helical stapled peptide to enter clinical trials. By mimicking the N-terminal domain of the p53 tumour suppressor protein, it binds with high affinity to both MDM2 and MDM4, the endogenous inhibitors of p53, in order to activate p53 signalling in cells [170] (Figure 4).

- Bcl-2

Furthermore, a stapled peptide based on Bcl-2 activity was created that displayed an increased helicity from 15.7% to 87.5%, half-life in vitro in serum from 3.1 to 29.4 h, and Kd value from 269 to 38.8 nM [171].

- β-catenin

**Figure 5:**
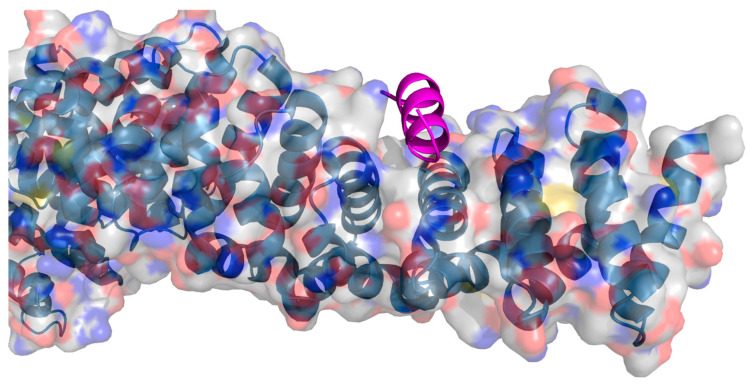
Crystal structure of the β-catenin in complex with stapled peptide inhibitor StAx-3–35R [172]. The receptor is depicted with a transparent surface coloured element, and the peptide appears in magenta as a graphic representation. PDB: 4DJS.

The interaction between β-catenin and T-cell factor (TCF), vastly reported as overexpressed in several cancers, was inhibited by the stapled peptide StAx-3–35R [172] (Figure 5). Challenges in inhibiting this PPI in vivo led to the alteration of this peptide with proteolysis-targeting chimeras (PROTACs), which maintained low levels of β-catenin for a much longer time period, suggesting a sustained β-catenin degradation [173].

- CYFIP

The protein Wiskott–Aldrich syndrome protein family member 1 (WASF1), which interacts with Cytoplasmic FMR1-interacting protein 1 (CYFIP1), promoting tumour cell movement, invasion, and metabolism, is persistently expressed at high levels in highly invasive prostate and breast cancer cells. The crystal structure revealed the interaction surface of this PPI, allowing the development of stapled peptides WAHM1 and WANM2 through a hydrocarbon stapling strategy using amino acid residues at positions 26 to 41 [174].

- Beclin-1

Beclin-1 mediates the cellular autophagy pathway, which is responsible for the degradation of the human epidermal growth factor 2 (HER2) in breast cancer and EGFR in NSCLC. The binding interface of the Beclin-1 homodimer was modelled by Yang et al., followed by the selection of a 15-residue fragment in the binding interface, which allowed the design of a stapled peptide that induced autophagy, contributing to the death of EGFR/HER2-driven cancer cells and exerting potent antiproliferative effects [175,176].

### 5.2. Backbone Modifications

Alterations in the backbone tend to change peptide properties more severely. The final sequences and, more importantly, the 3D structure, differ vastly from the original fragment. Still, the original peptide chain and the functional groups important to the binding site are retained; it is the unimportant sidechain moieties that are subjected to the modifications.

#### 5.2.1. Variation in Stereochemistry (D-Peptides)

**Figure 6:**
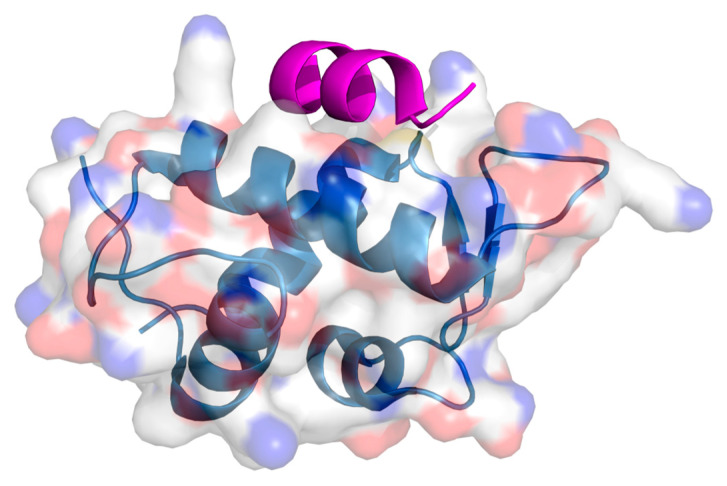
Crystal structure of the high-affinity D-peptide ligand of MDM2 [191]. The receptor MDM2 is shown with a transparent surface coloured element. The peptide is presented in the graphic representation in magenta. PDB: 3LNJ.

D-peptides are predominant for peptide-based therapeutics [65]. One notable advantage lies in their enhanced stability against proteolytic degradation, attributed to the stereochemical differences between D- and L-amino acids [177,178]. Being derived from natural proteins, L-peptides are usually more easily selected by the chiral proteases and quickly cleaved into their corresponding amino acid substituents. However, their D-counterparts, due to chirality mismatch with binding pockets, usually are poor substrates of endogenous enzymes. Hence, unlike L-amino acids, D-amino acids rarely act as the substrates of endogenous proteases, resulting in a resistance towards proteolysis [179]. Such approaches can easily prevent peptide inhibitors from rapid proteolytic degradation. Furthermore, this improved stability translates to an improved circulation in vivo, leading to extended half-lives compared to traditional L- peptides [180,181]. It has also been shown to enhance binding activity and specificity with receptor or target proteins [182,183]. However, the simple exchange of L- for D-amino acids can hinder the binding affinity or recognition as the sidechain orientations regarding the target become altered, thus impacting their efficacy as therapeutic agents [177,184]. This issue can be overcome by reversing the D-peptide sequence, hence restoring the L-amino acid chain angles. Retroinverso peptides have been used successfully to create peptides that exhibit a similar level of bioactivity to their L- counterparts. However, retroinversion also often fails, owing largely to the topological properties of helices, which impede binding from occurring [185,186]. Alternatively, mirror-image phage display involves synthesizing the target from D-amino acids and using it as bait for a randomized L-amino peptide library. Successful hits are reconstructed with D-amino acids [187]. The corresponding D-peptide, as the enantiomer of that L-peptide, can specifically bind to the natural L-target with high affinity, with some cancer-targeting D-peptide-based inhibitors having been obtained through this method [188,189,190,191,192]. Earlier research focused on peptides targeting crucial protein interactions, such as p53–MDM2 (Figure 6), VEGF–VEGF receptor, and PD-1–PD-L1 [191,192,193].

More recent developments include the following:

- HER2/HER3

Of particular interest, Pallerla et al. have successfully designed and synthesised a peptidomimetic to inhibit the HER2–HER3 interaction. HER2 receptors belong to the epidermal growth factor family of receptors, which are overexpressed in breast, lung, and ovarian cancers. To improve peptide stability, D-amino acids were introduced into the peptide, which resulted in an IC50 value in the nanomolar range in HER2-overexpressing cancer cell lines, as well as an increase in stability both in vitro and in vivo to 48 and 12 h, respectively [181].

- ERG

Wang et al. tested retroinversion-ERG inhibitory peptides (RI-EIP1) for ERG-mediated metastasis. Implantation of human prostate cancer cells onto a fertilized chicken embryo showed that RI-EIP1 treatment attenuated ERG-mediated transcription, chromatin recruitment, and protein–protein interactions and significantly reduced cancer cell invasion and proliferation and tumour growth, indicating its anti-metastatic effects [194].

- NRP1

**Figure 7:**
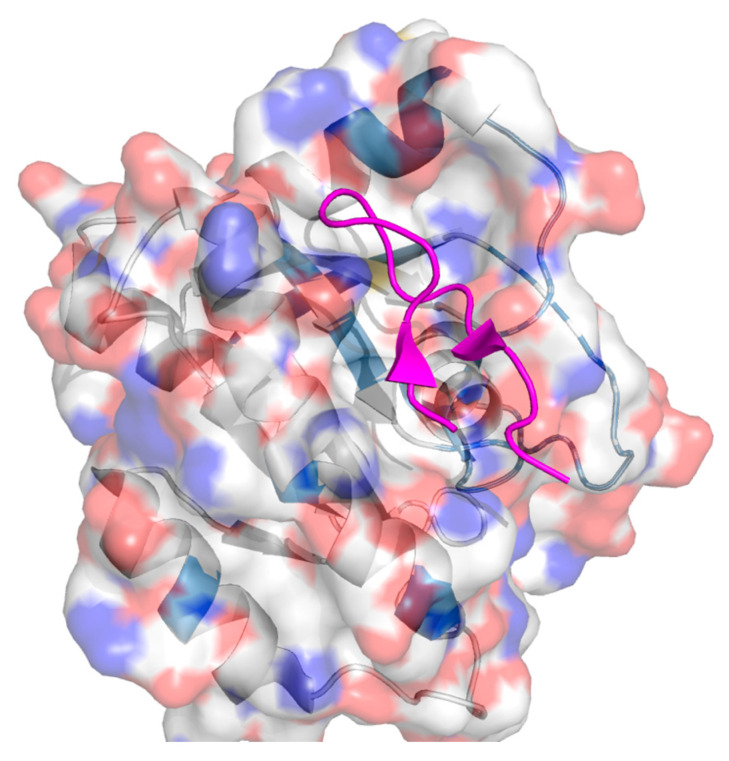
Crystal structure of K-Ras (G12D) GppNHp bound to cyclic peptide ligand KD2 [195]. PDB: 6WGN.

Furthermore, a D-peptide that targets NRP1, a cell–surface receptor overexpressed in lung cancer, and KRASG12D, a common oncogene, was developed using structure-based virtual screening. NKTP-3 showed a good biostability and a strong cellular uptake ability, and the group further demonstrated its antitumor effect both in vitro and in vivo [195] (Figure 7). Also, targeting NRP1 as a means to enter cancer cells is the work developed by Zhou et al. The development of a D-peptide that targets this overexpressed receptor on cancer cells as well as MDM2 has demonstrated strong anticancer activity to liver cancer cells in vitro and in vivo, with no apparent host toxicity [196].

- MYB-CBP/P300

In another study, Ramaswamy et al. developed a peptide mimetic of MYB residues 293–310 that interferes with the assembly of the MYB-CREB-binding protein/P300 complex. The developed peptidomimetic downregulated the MYB-bound BCL2 enhancer, leading to the downregulation of BCL2 expression and apoptosis of leukaemia cells. It also impeded leukaemia growth and extended survival of immunodeficient mice engrafted with primary patient-derived leukaemia cells [197].

#### 5.2.2. Extension of Backbone (Incorporation of β-Amino acids)

β-peptides are oligomers with beta-amino acids, which are commonly classified as foldamers-oligomers that form stable conformations in solution [198,199]. An additional advantage of these peptide classes is that they seem to have an unusually high resistance to degradation by proteases [130,200].

Two main approaches were defined for designing β-peptides, the exclusive use of β-residues and the combination of natural α-amino acids and constrained β-residues. The first consists of homologues of natural α-amino acids extended by one methylene group incorporated adjacent to the carboxylate or amine functional groups [201]. The latter is based on conformationally constrained monomers, often through a cycloalkane ring [198,202].

β-peptides commonly adopt a similar conformation to α-peptides despite the presence of an additional backbone carbon atom in each β-amino acid residue [203]. Nonetheless, replacing α-amino acid residues with β-analogues in the peptide sequence can lead to mismatch. Hence, a careful peptide design is implemented by selecting the appropriate placement of interacting residues. Moreover, due to the higher conformational stability, these improved peptides can be significantly shorter than α-peptides but still achieve a similar degree of folding [204].

Despite its potential, the incorporation of β-amino acids on peptide-based inhibitors for cancer targeting has seen limited reporting. Early studies produced several notable peptide-based PPI inhibitors, such as those targeting the Bcl-2–BH3 interaction, p53–HDM274, and p53-hDM2/hDMX [205,206,207]. Furthermore, starting with a 19-residue α-peptide, Haase et al. evaluated α to β replacements throughout the sequence. This enabled the identification of homologues containing up to approximately 30% of β-residues that retain significant affinity for VEGF, can block VEGF-stimulated proliferation, and display a substantial resistance to proteolysis [208]. Nevertheless, the momentum in developing these peptides, at least for cancer therapy, appears to have diminished over the past decade.

#### 5.2.3. Shift in the Sidechains to Nitrogen Atoms (Peptoids)

Peptoids are isomers of peptides in which all the sidechains are carried by the backbone nitrogens (N-substituted glycines) instead of on the α-carbons. This class of peptidomimetics is more flexible than peptides since intramolecular CO-HN hydrogen bonds are removed, thus being able to fold into helices that mimic the original peptide structure and function [209,210]. Almost any organic moiety can be incorporated into the peptide skeleton. By carefully selecting or modifying the sidechains, peptoids can be designed to better recognise target biomolecules, binding affinity, and protease stability while retaining the same functional density and backbone polarity [211,212]. This, however, also becomes the peptoid design’s main challenge. The appropriate selection for the surface of the peptide usually requires several attempts to place the key interacting residues in the effective position. The synthesis of peptoids is most commonly achieved by solid-phase synthesis, through the coupling of bromoacetic acid and substitution by a chosen amine, which can be performed on a solid resin support, allowing for the incorporation of a variety of substituents [213,214].

- PRMT1

Advancements in the field of peptoids include the inhibition of protein arginine methyltransferase 1 (PRMT1). PRMT1 contains a cysteine (Cys) within the active site. In order to improve binding and selectivity, Brekker et al. constructed a H4-16 peptoid by replacing a N-Arg residue, H4R3, with a chloracetamidine warhead, which is a known Cys modifier. The resulting peptoid induced apoptosis and autophagy in breast and colon cancer cells in a low micromolar range whilst not exhibiting any significant impact on nontumorigenic liver or normal human mammary epithelial cells [215].

- β-catenin

Another study designed a prostate cancer-targeting peptoid in silico that inhibits the β-catenin–TCF interaction, which is a pathway markedly overexpressed in a variety of cancers. In further assays, the peptoid exhibited potent antiproliferative effects in both prostate cancer cell lines and spheroids, as well as on a zebrafish model. The hypothesis that the introduction of this conformational constraint enforces steric and chemical complementarity to β-catenin is strongly supported by parallel testing with a linear analogue [216].

- Vimentin

To target vimentin, a cell surface–translocated cytoskeletal protein found in NSCLC, Zang et al. optimised a previously developed peptoid through an on-bead dimerization method to obtain homodimers or hetero-dimers by connecting two monomers at a C-terminus via a lysine linker. This translated into a 63-fold binding improvement over the parent peptoid along with potent anticancer activity characterised by an inhibitory effect on cell proliferation [217].

Collectively, these studies have provided significant examples of various chemical modifications that can be applied to peptides to enhance both their stability and bioavailability, as well as their therapeutic efficacy and specificity. By counteracting the limitations associated with peptide drugs, these advancements in peptide design further emphasise their potential as powerful agents in targeted cancer therapy.

## 6. Concluding Remarks and Future Perspectives

As crucial players in a broad range of biological and cellular processes, PPIs are among the most important targets in drug discovery. However, they have historically been underexplored due to their challenging biochemical and biophysical properties, namely their intractable interfaces, which are generally wide and flat and lack well-defined pockets for binding. The dysregulation of these interactions, especially in cancer cells, plays a pivotal role in oncogenesis by promoting tumour cell proliferation, invasion, and metastasis, thus highlighting the importance of targeting PPIs, or more specifically OncoPPIs, for effective cancer therapy.

Due to their unique advantages, peptide-based inhibitors have emerged as a powerful tool to address these PPI systems due to their unique advantages. Peptides exhibit high specificity and affinity for their targets and have lower toxicity and immunogenicity profiles than other therapeutics. Their structural similarity to natural ligands enables them to effectively disrupt specific PPI interfaces, offering a promising approach to cancer therapy. Despite these advantages, the development of peptide-based inhibitors is not without challenges as peptides characteristically have poor stability and a short half-life. However, advancements in peptide design and chemical modifications, such as cyclization and backbone alterations, have significantly improved their stability and bioavailability, thus enhancing their therapeutic potential. Of note, advancements in structural and computational biology have also further enabled the identification of novel peptide-based inhibitors with enhanced binding affinities, as well an in-depth exploration of the conformations of PPI systems.

Looking ahead, the focus on peptide-based inhibitors is poised to expand. Continuous advancements in technology, the integration of high-throughput screening methods, and comprehensive OncoPPI databases will further facilitate the identification and characterisation of novel peptide-based inhibitors. This potential for future research underscores the importance and promise of peptide-based inhibitors in the field of cancer therapy.

In conclusion, the unique ability of peptide-based inhibitors to disrupt crucial OncoPPIs with high specificity and reduced side effects positions them as ideal candidates for cancer therapy. With continued research in this field, peptide-based inhibitors are poised to become a cornerstone in cancer therapy, offering targeted, effective, and less toxic treatment options that will ultimately improve cancer patient outcomes. This potential highlights the importance of further research and development in this area.

## Figures and Tables

**Table 1:** FDA-approved peptide therapeutics since 2005.

Peptide	Approval Year	Molar Mass (g/mol)	Target	Half-Life	Administration	Therapeutic Application
Exenatide [17]	2005	4186.63	GLP-1	2.4 h	SC	Type 2 diabetes mellitus
Pramlintide [18]	2005	3949.44	Calcitonin receptor	48 min	SC	Type 1 and 2 diabetes mellitus
Anidulafungin [19]	2006	1140.254	beta-1,3-D-glucan synthase	40–50 h	IV, O	Fungal infections: Candida infections, Aspergillus infections, and esophageal candidiasis
Lanreotide [20]	2007	1096.33	SSTR	22 days	SC	Neuroendocrine tumours and acromegaly
Degarelix [21]	2008	1632.29	GNRH receptor	41.5–70.2 days	SC	Prostate cancer
Icatibant [22]	2008	1304.54	Bradykinin B2 receptor	1–2 h	SC	Hereditary Angioedema
Liraglutide [23]	2009	3751.262	GLP-1	13 h	SC	Type 2 Diabetes Mellitus
Mifamurtide [24]	2009	1237.518	NOD2	18 h	IV	High-grade resectable non-metastatic osteosarcoma
Romidepsin [25]	2009	540.69	Histone deacetylase	3 h	IV	Cutaneous T-cell lymphoma (CTCL) or/and peripheral T-cell lymphoma (PTCL)
Telavancin [26]	2009	1755.65	Peptidoglycan	8 ± 1.5 h	IV	Complicated skin and skin structure infections (cSSSI) caused by gram-positive bacteria like methicillin-susceptible or -resistant Staphylococcus aureus
Tesamorelin [27]	2010	5005.76	GnRH receptors	38 min	SC	Reduction of lipodystrophy in HIV-infected patients
Telaprevir [28]	2011	679.863	NS3/4A viral protease	4 h	O	Chronic Hepatitis C genotype 1 infection in adults
Teduglutide [29]	2012	3752.13	GLP-2	1.3 h	SC	Short bowel syndrome (SBS)
Linaclotide [30]	2012	1526.73	GC-C receptor		O	Irritable bowel syndrome (IBS)
Pasireotide [31]	2012	1047.227	SSTR	12 h	SC, IM	Cushing’s disease
Carfilzomib [32]	2012	719.924	Proteosome	≤1 h	IV	Multiple myeloma
Lixisenatide [33]	2013	4858.56	GLP-1	3 h	SC	Type 2 Diabetes mellitus
Afamelanotide [34]	2014	1646.874	MCR	30 min	SC	Prevention of phototoxicity in adult patients with erythropoietic protoporphyria (EPP)
Dalbavancin [35]	2014	1816.7	Peptidoglycan	346 h	IV	Adult patients with acute bacterial skin and skin structure infections (ABSSSI)
Oritavancin [36]	2014	1793.12	Peptidoglycan	245 h	IV	Skin infections
Ombitasvir [37]	2014	894.12	NS5A	21–25 h	O	Genotype 4 chronic hepatitis C virus (HCV) infection
Etelcalcetide [38]	2016	1048.26	CaSR	3–4 days	IV	Secondary hyperparathyroidism (HPT)
Abaloparatide [39]	2017	3960.657	PTH1R	1.7 h	SC	Osteoporosis
Plecanatide [40]	2017	1681.89	GC-C receptor		O	Chronic idiopathic constipation (CIC)
Angiotensin II [41]	2017	1046.197	ATR	<1 min	IV	Sepsis and septic Shock
Semaglutide [42]	2017	4113.641	GLP-1	168 h	SC, O	Type 2 diabetes mellitus
Lutetium (Lu)^177^-Dotatate [43]	2018	1609.55	SSTR	71 h	IV	Somatostatin receptor-positive gastroenteropancreatic neuroendocrine tumors
Bremelanotide [44]	2019	1025.182	MCR	2.7 h	SC	Hypoactive sexual desire disorder
Setmelanotide [45]	2020	1117.3	MCR	11 h	SC	Chronic weight management of obesity
Difelikefalin [46]	2021	679.863	kappa opioid receptor	23–31 h	IV	Moderate-to-severe pruritus associated with chronic kidney disease
Voclosporin [47]	2021	1214.646	Calcineurin	30 h	O	Lupus nephritis
Odevixibat [48]	2021	740.93	IBAT	2.36 h	O	Pruritus in patients with progressive familial intrahepatic cholestasis.
Vosoritide [49]	2021	4102.78	NPR-B	21–27.9 min	SC	Pediatric patients with achondroplasia
Tirzepatide [50]	2022	4813.53	GLP-1	5 days	SC	Type 2 diabetes mellitus
Trofinetide [51]	2023	315.14	IGF-1R	1.5 h	O	Rett syndrome
Motixafortide [52]	2023	2159.6	CXCR4	2 h	SC	Multiple myeloma
Nirmatrelvir [53]	2023	499.5	M^pro^	6.05 h	O	Mild-to-moderate COVID-19
Zilucoplan [54]	2023	3562.23	Complement protein C5	172 h	SC	Myasthenia gravis
Danicopan [55]	2024	580.4	Complement factor D	7.9 h	O	Extravascular hemolysis in patients that have paroxysmal nocturnal hemoglobinuria

Abbreviations: ATR: angiotensin II receptors; CaSR: calcium-sensing receptor; CXCR4: C-X-C Motif Chemokine Receptor 4; GC-C: guanylate cyclase-C; IGF-1R: Insulin-like Growth Factor-1 Receptor; GnRH: growth hormone-releasing hormone; GLP-1: glucagon-like peptide-1; IBAT: ileal bile acid transporter; IM: intramuscular; IV: intravenous; MCR: melanocortin receptor; NOD2: nucleotide-binding oligomerization domain-containing protein 2; NPR-B: natriuretic peptide receptor B; O: oral; PTH1R: parathyroid hormone-related receptor-1; SC: subcutaneous; SSTR: somatostatin receptor.

## Data Availability

All data presented in this review are available in the manuscript.

## Abbreviations

The following abbreviations are used in this manuscript:

Ala	Alanine
Asp	Aspartic Acid
Bcl-2	B-cell lymphoma-2
Bcl-xL	B-cell lymphoma-extra large
BRCA1	Breast cancer type 1
CLL	Chronic lymphocytic leukaemia
CYFIP1	Cytoplasmic FMR1-interacting protein 1
Cys	Cysteine
CXCR4	C-X-C Motif Chemokine Receptor 4
GC-C	Guanylate cyclase-C
EGFR	Epidermal growth factor receptor
FDA	Food and Drug Administration
Gly	Glycine
HBS	Hydrogen bond surrogate
HDM2	Human MDM2
HER2	Human epidermal growth factor 2
His	Histidine
IBAT	Ileal bile acid transporter
IGF-R1	Insulin-like Growth Factor-1 Receptor
GnRH	Growth hormone-releasing hormone
GLP-1	Glucagon-like peptide-1
IM	Intramuscular
IV	Intravenous
KRAS	Kristen Rat Sarcoma Viral oncogene homolog
mAbs	Monoclonal antibodies
MCR	Melanocortin receptor
MW	Molecular weight
NOD2	Nucleotide-binding oligomerization domain-containing protein 2
NPR-B	Natriuretic peptide receptor B
NSCLC	Non-small cell lung carcinoma
O	Oral
OncoPPIs	Oncogenic PPIs
PK	Pharmacokinetic
PPIs	Protein–protein interactions
PRMT1	Protein arginine methyltransferase 1
PROTAC	Peptide with proteolysis-targeting chimera
PTH1R	Parathyroid hormone-related receptor-1
Ras	Rat sarcoma virus
RI-EIP1	Retroinverso-ERG inhibitory peptides
RTK	Receptor tyrosine kinase
SC	Subcutaneous
SMDs	Small-molecule drugs
Sos	Son of sevenless
SSTR	Somatostatin receptor
TCF	T-cell factor
Trp	Tryptophan
Tyr	Tyrosine
WASF1	Wiskott–Aldrich syndrome protein family member 1
